# Biodirected Synthesis of Silver Nanoparticles Using Aqueous Honey Solutions and Evaluation of Their Antifungal Activity against Pathogenic *Candida* Spp.

**DOI:** 10.3390/ijms22147715

**Published:** 2021-07-19

**Authors:** Grzegorz Czernel, Dominika Bloch, Arkadiusz Matwijczuk, Jolanta Cieśla, Monika Kędzierska-Matysek, Mariusz Florek, Mariusz Gagoś

**Affiliations:** 1 Department of Biophysics, University of Life Sciences in Lublin, Akademicka 13, 20-950 Lublin, Poland; arkadiusz.matwijczuk@up.lublin.pl; 2 Department of Cell Biology, Institute of Biology and Biochemistry, Maria Curie-Skłodowska University, Akademicka 19, 20-033 Lublin, Poland; dominika.bloch@poczta.umcs.lublin.pl (D.B.); mariusz.gagos@poczta.umcs.lublin.pl (M.G.); 3 Institute of Agrophysics, Polish Academy of Sciences, Doświadczalna 4, 20-290 Lublin, Poland; j.ciesla@ipan.lublin.pl; 4 Department of Quality Assessment and Processing of Animal Products, University of Life Sciences in Lublin, Akademicka 13, 20-950 Lublin, Poland; monika.matysek@up.lublin.pl (M.K.-M.); mariusz.florek@up.lublin.pl (M.F.); 5 Department of Biochemistry and Molecular Biology, Medical University of Lublin, 20-093 Lublin, Poland

**Keywords:** silver nanoparticles, honey, aggregation, antifungal activity, zeta potential, molecular spectroscopy

## Abstract

Silver nanoparticles (AgNPs) were synthesized using aqueous honey solutions with a concentration of 2%, 10%, and 20%—AgNPs-H2, AgNPs-H10, and AgNPs-H20. The reaction was conducted at 35 °C and 70 °C. Additionally, nanoparticles obtained with the citrate method (AgNPs-C), while amphotericin B (AmB) and fluconazole were used as controls. The presence and physicochemical properties of AgNPs was affirmed by analyzing the sample with ultraviolet–visible (UV–Vis) and fluorescence spectroscopy, scanning electron microscopy (SEM), and dynamic light scattering (DLS). The 20% honey solution caused an inhibition of the synthesis of nanoparticles at 35 °C. The antifungal activity of the AgNPs was evaluated using opportunistic human fungal pathogens *Candida albicans* and *Candida parapsilosis*. The antifungal effect was determined by the minimum inhibitory concentration (MIC) and disc diffusion assay. The highest activity in the MIC tests was observed in the AgNPs-H2 variant. AgNPs-H10 and AgNPs-H20 showed no activity or even stimulated fungal growth. The results of the Kirby–Bauer disc diffusion susceptibility test for *C. parapsilosis* strains indicated stronger antifungal activity of AgNPs-H than fluconazole. The study demonstrated that the antifungal activity of AgNPs is closely related to the concentration of honey used for the synthesis thereof.

## 1. Introduction

Candidiasis is one of the most common infections in the world. The disease is caused by the *Candida albicans* and *Candida* non-*albicans* groups. *C. albicans* is the most prevalent yeast species in oral, genital, and skin infections (≥90%). It is also responsible for severe systemic mycoses in immunosuppressed patients. *Candida parapsilosis* is an important factor in catheter-related yeast bloodstream infections. The main factor in *C. parapsilosis* virulence is its ability to colonize artificial surfaces, which is associated with the ability of this yeast to form biofilms. The species is regarded as an important etiological factor of nosocomial infections in patients undergoing medical procedures that require the use of vascular catheters and tubing [1]. The administration of a wide range of antifungal drugs (e.g., amphotericin B and azoles) has been found to increase drug resistance [2]. In this aspect, nanotechnology offers a possibility of development of new therapeutic agents. Due to their unique physicochemical properties, silver nanoparticles (AgNPs) are increasingly being used in science and technology [3]. With its strong antimicrobial properties, nanosilver is applied in medicine in fabrics, coatings, implants, and dressings, as well as in the treatment of wounds and burns [4]. It is also used for disinfection of water and in elements of medical devices and instruments [5]. The controlled synthesis of metal nanoparticles of a defined morphology is important for fields such as biochemistry, catalysis, and medicine [6] Nanomaterials also represent a promising novel class of materials to be used as antimicrobial agents [7,8].

Currently, there are several known methods of AgNP synthesis—physical, chemical, and biological. Green synthesis is the most suitable method in biomedicine for safety reasons [9]. In this method, nanoparticles are synthesized using natural products which themselves often have antimicrobial properties, thereby enhancing the effect of the nanoparticles through synergistic or additive effects. For these reasons and due to ecological concerns, the method of green synthesis is currently gaining in popularity. For the green synthesis of nanoparticles to occur, it is necessary to provide Ag^+^ ions with a biological reducing agent. In most cases, reducing agents also act as stabilizers and coating agents. AgNP synthesis in biological systems takes place in the presence of numerous organic compounds such as carbohydrates, protein, free amino acids, enzymes, phenolic compounds, terpenoids, and alkaloids, with the ability to donate electrons required for reduction of Ag^+^ ions to Ag^0^. The synthesis of AgNPs can also be triggered with the use of aqueous honey solutions, where the sugars contained therein, mainly mono- and disaccharides, act as the key reducing agents [10,11].

Honey is known as an excellent remedy for healing wounds since ancient times. As reported recently, honey may be suggested as a potential source of natural antimicrobial agents against *C. albicans*, i.e., the most common fungal pathogen in humans [12]. The use of honey for AgNP synthesis offers numerous benefits due to its inherent antimicrobial properties.

Only sparse information is available on the antifungal activity of AgNPs biosynthesized using honey [13,14,15]. Therefore, in this study, we investigated their activity against two opportunistic pathogenic *C. albicans* and *C. parapsilosis* species. Additionally, the impact of the concentration of honey used for the synthesis of nanoparticles on their antifungal activity was analyzed.

## 2. Result

### 2.1. Biosynthesis and Spectroscopic Characterization of AgNPs

The synthesis of nanoparticles with the citrate reduction method is described in Section 5 and in our previous publication [16]. The synthesis of nanoparticles using aqueous honey solutions (AgNPs-H) can be confirmed by measuring the surface plasmon resonance (SPR) band using electronic absorption spectroscopy. The ultraviolet–visible (UV–Vis) spectrum of the reaction mixture has an absorption peak at 400 nm; this evidences the presence of SPR of nanoparticles. A single SPR band indicates that nanoparticles have a spherical shape [17,18]. During the first stage of the experiment, the synthesis of AgNPs was conducted at temperature of 35 °C in aqueous honey solutions at three concentrations: 2%, 10%, and 20% (AgNPs-H2, AgNPs-H10, and AgNPs-H20). The precursor (AgNO_3_) concentration in each sample was 1 mM, pH 9.5. The reducing sugars glucose and fructose naturally occurring in honey were the reducing factor, and proteins were the capping factor [19]. An alkaline environment is necessary for AgNP synthesis; a high pH value facilitates the opening of the glucose ring by splitting the α-proton from the oxygen ring, and metal ions oxidize glucose to gluconic acid. Initially, the formation of nanoparticles was confirmed by the visible change in the color of the synthesis mixture from pale yellow to light brown. This eventual color change is related to the SPR of nanosilver, which results in the relevant UV–Vis spectrum.

**Figure 1:**
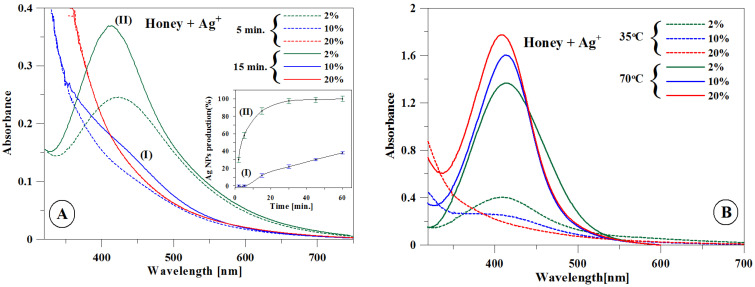
(**A**) Ultraviolet–visible (UV–Vis) spectra of silver nanoparticles (AgNPs) recorded after 5 (dotted line) and 15 min (solid line) for the 2%, 10%, and 20% concentrations at 35 °C. Inset: Changing rate of production of AgNPs measured by changes in absorbance at 400 nm with time. (**B**) Spectra for the 2%, 10%, and 20% concentrations recorded at 35 °C (dotted line) and 70 °C (solid line) after 1 h.

Figure 1A presents the UV–Vis spectra for the aqueous honey solutions with concentrations of 2%, 10%, and 20% at 5 and 15 min after the addition of Ag^+^ ions at 35 °C. In the 2% solution, a characteristic SPR band centered at approximately 400 nm was observed after 5 min. In the 10% and 20% solutions, no spectral changes that could indicate the formation of nanostructures were observed.

After 15 min, a relatively wide low-intensity band was registered for the 10% solution. In the case of the 20% solution, even after 60 min, no spectral changes that would indicate the presence of AgNP synthesis in the relevant combination of concentration and temperature were observed. A change in the rate of AgNP formation relative to the reaction speed is presented in the inset in Figure 1A. Since the maximum SPR is directly proportional to the concentration of AgNPs, the above analysis allows the estimation of the relative synthesis efficiency in the described system. Figure 1B presents the SPR spectra for AgNPs-H2, AgNPs-H10, and AgNPs-H20 at the temperatures of 35 °C and 70 °C after 60 min. This was the minimum time frame required for the full synthesis of nanoparticles to take place. In the case of the AgNPs-20 synthesis at the temperature of 70 °C, a band with the highest intensity was recorded. The AgNPs-H2, AgNPs-H10, and AgNPs-H20 produced bands with the maxima at 414 nm, 413 nm, and 407 nm, respectively. The full width calculated for the final bands at half-maximum (51 nm, 40 nm, and 43 nm for AgNPs-2, AgNPs-10, and AgNPs-20, respectively) indicates that the highest uniformity was observed for AgNPs obtained using the 10% honey solution [20].

**Figure 2:**
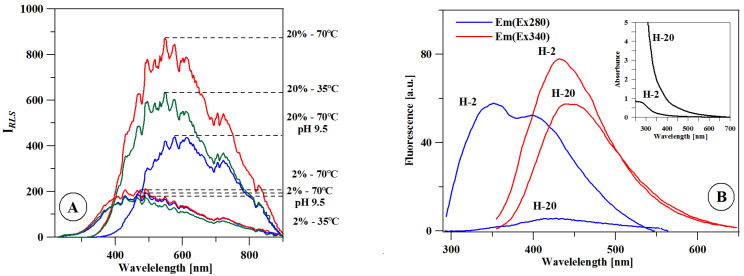
(**A**) Resonance light scattering (RLS) spectra of pure honey in different temperature, concentration, and pH conditions. (**B**) Fluorescence emission spectra of honey at the 2% and 20% concentrations. Inset: Electronic absorption spectra for analogical samples.

Figure 2A presents the resonance light scattering (RLS) spectra for the 2% (H-2) and 20% (H-20) solutions of pure honey at the temperatures of 35 °C, 70 °C, and 70 °C after increasing the pH value to 9.5. The initial pH of the solutions was 5.5. For the H-2 concentration, the increase in the temperature and the change in pH caused no significant changes in the intensity of the RLS spectrum. For H-20, on the other hand, the intensity of the RLS band increased with the temperature rise to 70 °C but dropped at the higher pH.

Figure 2B presents the emission spectra for the H-2 and H-20 solutions, as well as the respective electronic absorption spectra (inset A). As shown, the higher absorbance of the H-20 sample is correlated with the lower intensity of emission spectra, particularly with regard to the band under excitation at 280 nm. The effect may be associated with the fluorescence-quenching effect of the concentration or the lower availability of fluorophores (e.g., amino acids, polyphenols) [21,22] in the H-20 sample due to aggregation.

### 2.2. Scanning Electron Microscopy

**Figure 3:**
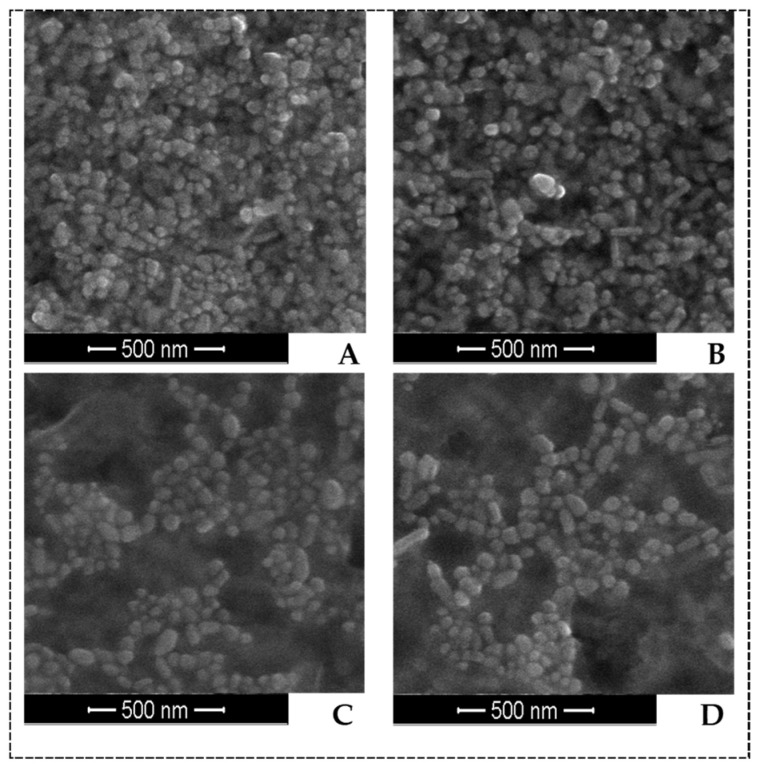
Scanning electron microscopy micrographs of AgNPs (scale 500 nm): (**A**) AgNPs-C, (**B**) AgNPs-H2, (**C**) AgNPs-H10, and (**D**) AgNPs-H20; synthesis at 70 °C.

Scanning electron microscopy (SEM) was employed to characterize the size, shape, and morphology of AgNPs synthesized at 70 °C. An SEM image of AgNPs is shown in Figure 3. It elucidates the formation of isotropic and nearly spherical nanoparticles, which is in agreement with the shape from the SPR band in the UV–Vis spectra. The images also showed a small number of rod-shaped nanoparticles. These structures can be formed during synthesis, as acquisition of all nanoparticles with a specific shape and size is quite difficult. This is related to the fact that the balance between nucleation and growth is difficult to regulate during the synthesis of nanoparticles in aqueous solutions [17]. The average particle size measured in the images was 42–55 nm (AgNPs-C), 55–75 nm (AgNPs-H2), 55–70 nm (AgNPs-H10), and 66–80 nm (AgNPs-H20).

### 2.3. Dynamic Light Scattering Study

The results of the particle size measurement (dynamic light scattering method), zeta potential (ZP; laser doppler electrophoresis method) of dispersed particles, and the electrolytic conductivity (EC) of the samples are summarized in Table 1. Moreover, since the samples after the AgNP synthesis still contained some ingredients of the initial dispersing medium used (i.e., citrate or honey solution), the properties of honey solutions at the concentrations of 2%, 10%, and 20% (i.e., H2, H10 and H20, respectively) and the citrate solution (C) are shown in Table 1.

In the case of the citrate solution, the intensity of scattered light was too low to obtain reliable results. In the systems based on the honey solutions, Z_ave_ decreased and PdI increased after the AgNP synthesis, confirming the presence of small particles next to the large ones. The values of PdI higher than 0.5 pointed out non-homogeneity of the samples in terms of the size of dispersed particles (reflected as a non-monomodal particle size distribution, i.e., the dependence of the percentage of scattered light intensity on the particle diameter). In this case, the analysis of the peak location would be more suitable for characterization of the sample than the use of the Z_ave_ value. For all the honey concentrations used, the AgNP synthesis resulted in the appearance of a new peak with the maximum at about 30 nm next to the peak determined for the honey solutions (at the hydrodynamic diameter higher than 100 nm). It should be stressed that the ingredients of the aqueous solution of honey may form assemblies whose size decreases with dilution [23]. For better determination of differences between the samples after and before the AgNP synthesis, differential particle size distributions were obtained, considering only the effects which led to an increase in the percentage of scattered light intensity [24]. Accordingly, it was observed that the AgNP synthesis resulted in an increase in the peak height at a particle size of 24 and 142 nm for AgNPs-H2, 24 and 164 nm for AgNPs-H10, and 28 and 220 nm for AgNPs-H20.

The zeta potential distributions were monomodal for all samples studied. The AgNP synthesis did not generate new peaks. Compared to the honey solutions, an increase in the percentage of scattered light intensity was observed at −16 mV for AgNPs-H2 and −30 mV for both AgNPs-H10 and AgNPs-H20.

### 2.4. Determination of Minimal Inhibitory Concentration

Due to the low efficiency of the synthesis carried out at 35 °C, nanoparticles obtained at a temperature of 70 °C were used in the analyses of antifungal activity. The antifungal activity of AgNPs-H against two *Candida* spp. strains was assessed using the broth microdilution method. The activity of AgNPs-H was compared with that of the nanoparticles obtained with the citrate reduction method (AgNPs-C). The minimal inhibitory concentration (MIC) values of four colloidal suspensions of AgNPs against *Candida* spp. cells are presented in Table 2. Both species were shown to be susceptible to very low concentrations of AgNPs-H2 and AgNPs-C. The lowest MIC values were obtained for AgNPs-H2, i.e., 0.5 μg/mL, in both strains. The AgNPs-H10 and AgNPs-H20 solutions did not exhibit any antifungal activity in the tested concentration range. The sodium citrate and honey solutions applied in the tested concentration range did not have any antifungal activity against the *Candida* spp.

**Figure 4:**
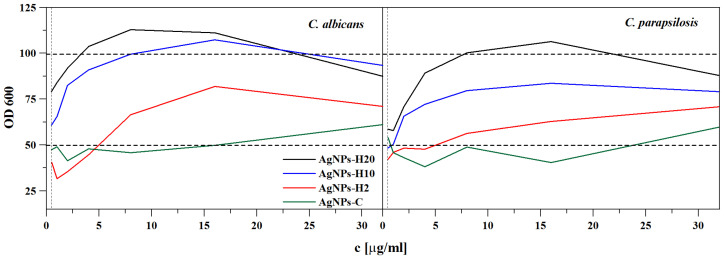
Optical density (OD) at 600 nm as a function of the silver nanoparticle AgNP concentration.

Figure 4 shows the dependence of the optical density (OD) 600 value of the analyzed suspensions on the concentration of nanoparticles. AgNPs-H20 applied in the concentration range of 16–4 µg/mL and 16–8 µg/mL stimulated the growth of *C. albicans* and *C. parapsilosis*, respectively. The same effect of AgNPs-H10 used in the range of 16–8 µg/mL was observed in the case of *C. albicans*. Pure honey solutions, H-20 and H-10 showed a similar fungal stimulation effect to AgNPs-H20 and AgNPs-H10, respectively.

### 2.5. Disc Diffusion Assay

**Figure 5:**
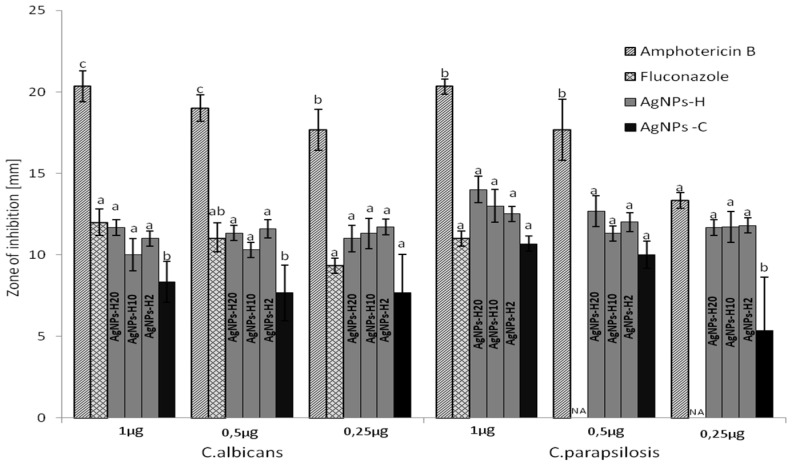
Graphical presentation of zone of inhibition ZOI against *Candida* strains. Moderate inhibition zone (6–9 mm); strong inhibition zone (10–14 mm); very strong inhibition zone (15–18 mm); NA (no activity). Each result is shown as a mean value with SD of results from two cultures in three replications. The same letter means that the differences between the data compared are not statistically significant. Different letters (a, b, and c) mean that the data compared are statistically different at *p* < 0.05.

The results for amphotericin B (AmB), fluconazole, AgNPs-H20, AgNPs-H10, AgNPs-2, and AgNPs-C are presented below. The zone of inhibition (ZOI) in millimeters of respective discs loaded with different concentrations of the antifungal agents was determined, and the results are shown in Figure 5. No significant differences were observed between the nanoparticles obtained in the different concentrations of honey in the disc diffusion assay.

The diameters of the inhibition zones of the analyzed AgNP solutions ranged from 8 to 14 mm. AgNPs-H20 (1 μg) caused the greatest inhibition of *Candida* strains. The inhibitory effect of AgNPs-H was significantly higher than that of AgNPs-C against *C. albicans* in the 1 µg and 0.5 µg variants and against *C. parapsilosis* at the 0.25 µg concentration. In the case of the standard antifungal drugs used in the study, the inhibition zone ranged from 12 to 20 mm and from 0 to 12 mm in the case of AmB and fluconazole, respectively. AgNPs-H and AgNPs-C exhibited lower activity against *C. albicans* than AmB. The activity of AgNPs-H against *C. albicans* was comparable to that of fluconazole. However, fluconazole administered at the concentrations of 0.5 µg and 0.25 µg had no activity against *C. parapsilosis*.

## 3. Discussion

In the first stage, the AgNPs-H synthesis was carried out at a temperature of 35 °C (Figure 1). In these thermal conditions, the course of the synthesis was atypical in the case of some concentrations of honey. It was expected that the higher content of reducing agents in the 20% solution would enhance the reaction rate. Instead, the reaction did not take place at all in this concentration and proved to be highly inefficient in the 10% solution. In the subsequent part of the study, the influence of temperature on AgNPs-H synthesis was analyzed with a focus on the highest concentration of honey in the solution. The analysis of the dependence of the reaction efficiency on the temperature [25] showed that the observed effect may be related to the aggregation of honey components. In the complex matrix of honey, macromolecules self-associate with colloidal particles at a critical concentration driven by the viscosity of honey. The detailed chemical composition of colloidal honey particles has not been fully identified [26]. As shown in previous studies, colloids in honey constituted from 0.1% to 1% of honey weight and proteins constituted 54% [27,28]. Furthermore, it was observed that large colloidal particles of dark and medium honeys exhibited remarkable colloidal stability [23] although the dilution with water was increased from 16- to 32-fold. The impact of temperature on nanoparticle synthesis in the 20% concentrated honey solution can be explained by the formation of aggregated structures as a result of protein–carbohydrate interaction [29,30] and occurrence of polyphenol–protein complexes in honey. For example, noncovalent protein–protein interactions between glycosylated MRJP1 monomers lead to the formation of a large 350 to 420 Da heterohexamer apisin [31], whereas both noncovalent and covalent interactions between protein and polyphenols lead to the formation of protein–polyphenol complexes [32] and melanoidins [33]. It should be noted that these types of interactions leading to the formation of aggregates can significantly reduce the availability of reducing components in the honey solution during synthesis at low temperature [29]. In the case of lower concentrations, there are lower amounts of reducing substances which are, however, more readily available. As the concentration increases, associates of organic compounds and crystalline structures of compounds responsible for the reduction of silver ions may form. A temperature increase to 70 °C causes the breakage of bonds, particularly of the van der Waals type and hydrogen bonds, thereby initiating the processes of monomerization. Furthermore, increased temperature also significantly accelerates the reaction of Ag^+^ ion reduction.

The resonance light scattering (RLS) technique is commonly used for a variety of analytical measurements [34]. It can also be applied to study intermolecular interactions, particularly with regard to chromophoric aggregation processes between molecules [35]. The absence of changes in H-2 indicates no influence of temperature and pH on the level of aggregation; hence, AgNP synthesis occurs in this concentration at both 35 °C and 70 °C. The higher synthesis efficiency observed in this solution at the increased temperature (Figure 2A) is mostly due to the increased rate of the reaction rather than an altered level of aggregation of the components. Entirely different results were obtained for the H-20 solution. Compared to H-2, the initial band was characterized by significantly greater intensity, and its maximum was shifted by approximately 10 nm in the long-wave direction.

The observed spectral differences indicated that aggregated forms (e.g., proteins) of various sizes were present in these solutions. As mentioned above, the technique allows for the observation of certain aggregate forms, particularly those resulting from chromophoric interactions within a certain size range. This means that, in most cases, it is impossible to observe small dimer and trimer structures or structures of very large sizes [36]. This may explain the increased intensity of the scattering signals in the H-20 solutions after the temperature increase. Very large structures strongly aggregated at 35 °C may not generate RLS signals. However, once the temperature is increased, the large structures fall apart and smaller structures emerge, to which the increased band intensity could be attributed. Only once the pH is increased to 9.5 does the actual disaggregation take place, which accounts for the decreased intensity of the RLS band. Summing up, this unexpected effect of increasing the intensity of RLS spectra with temperature may be related to disaggregation of bee pollen at higher temperatures. Pollen contains, on average, 20% protein and has the form of grains with quite varied sizes (2.5–250 µm) [37]. Changes in the size of these structures with increasing temperature may have a significant effect on changing the intensity of RLS bands. The RLS band may be related to light scattering at the excitonic levels of interacting chromophores of compounds concentrated in the solution. The bathochromic shift of the RLS spectra in relation to the fluorescence emission spectra proves the independence of these bands from the autofluorescence of compounds contained in the solution. This indicates their relationship with chromophore aggregated molecular systems.

The results of the antifungal activity against *C. albicans* clearly indicated that fungal growth was inhibited already at concentrations of 2 µg/mL of AgNPs-C and 0.5 µg/mL of AgNPs-H2. These findings correlate well with results reported by other authors [38], who analyzed nanoparticles biosynthesized in other systems, although higher MIC values were obtained in some studies (60 µg/mL–30 µg/mL) [39,40]. The basic mechanisms of the antifungal activity of AgNPs consist of interactions with microbial membrane proteins and DNA, production of reactive oxygen species, and interactions with the cell wall or membrane, leading to disturbances in the membrane potential [41]. Interactions between the surface of fungal cell membrane and nanoparticles are a complex problem related to the properties of the cell membrane, nanoparticles, and the dispersing medium used. Functional groups present on the surface of nanoparticles (e.g., proteins) determine many essential properties of nanomaterials, e.g., solubility or surface charge, which influence the interaction of nanomaterials with the cell surface, including internalization of nanoparticles [4,42,43,44]. The net negative surface electrical charge of the cell membrane facilitates electrostatic attraction with the positively charged nanoparticles or with the functional groups of macromolecules present in the solution [45]. However, the surface of the cell membrane is usually nonhomogeneous in terms of the electrical charge distribution. The net electrical charge of the cell surface varies among *Candida* species [46]. In our study, the hydrodynamic diameter of AgNPs-H was higher than that of AgNPs-C. The literature describes a correlation between antimicrobial activity and the size of nanoparticles [47,48,49]. It has been shown that the activity generally increases with a decrease in nanoparticle diameter. In contrast, the authors of the study [50] demonstrated that antifungal activity does not depend on the nanoparticle size. This was confirmed by our research showing that AgNPs-C with a smaller diameter exhibited less potent antifungal activity. These results may be associated with the impact of the surface charge, as well as the composition and structure of the electrical double layer around AgNPs [51]. There are only limited data on the zeta potential of stabilized AgNPs analyzed for antimicrobial activity [38,52,53,54,55]. Such activity of AgNPs synthesized using maltose was investigated by Çulha et al. [56]. The zeta potential value determined by these authors was close to that obtained for AgNPs-H, while the zeta potential of AgNPs synthesized using honey from *Z. spina-christi* and *A. gerrardii* was close to zero [57]. In the present study, the values of the zeta potential of both AgNPs-C and AgNPs-H were negative. However, the absolute value of the zeta potential of AgNPs-H was lower (less negative) than that of AgNPs-C (Table 1), probably resulting in weaker repulsion from the cell membrane. This suggests that the AgNPs-H were less repelled from the membrane surface than the AgNPs-C. Additionally, the *Candida* species were different, considering their surface charge. It was shown that the value of zeta potential of *C. parapsilosis* was more negative than that of *C. albicans*. [46]. The presence of flavonoids contained in honey may also be an additional factor responsible for the increasing activity of AgNPs-H [12]. This effect may result from the synergistic action of nanoparticles and phenolic compounds contained in honey.

In the case of AgNPs-H10 and AgNPs-H20, no activity and even stimulated fungal growth were observed. One of the mechanisms of the lower activity of the solutions with the higher honey content may be related to the fact that honey components are a substrate for *Candida* growth. The honey concentrations in the case of AgNPs-H20 applied in the range of 16–8 µg/mL (Figure 4) were 3.2–1.65%, and stimulation of *C. albicans* growth was observed. A similar effect was described in [21], where the authors analyzed the effect of honey concentration on its antifungal activity. The analysis of the OD curves related to fungal growth revealed a clear peak at the 2.5% and 5% concentrations. The additional carbohydrates, as in the case of the sugar control, stimulated *C*. *albicans* growth to an optimal level. Above this concentration, the inhibitory factors present in the honey partially inhibited the growth of the fungi. The analysis of the ZOI revealed no significant relationship between the activity and the concentration of honey used for the synthesis of nanoparticles. The components of the sample placed on the dish surface did not diffuse; hence, there was no stimulatory effect of the honey components on fungal growth. Additionally, evaporation leads to natural concentration of the sample. The concentration increases to a level where honey inhibits fungal growth. This is in line with other reports [58], where the addition of undiluted honey to the culture medium resulted in complete growth inhibition. The addition of 50% and 20% of honey into the fungal growth medium resulted in sparse growth and good growth, respectively. This may mean that there is a range of concentrations in which honey can serve as a nutrient and promote microbial growth. AgNPs-H were also highly active in the disc diffusion test, although their activity was lower than that of (AmB). Noteworthy, this antibiotic is a highly effective antifungal agent used as the gold standard with which newer agents are compared [59,60]. In comparison with fluconazole, the AgNPs-H activity was similar in the case of *C. albicans* and better than the drug against *C. parapsilosis*.

## 4. Conclusions

The present study focused on the synthesis of AgNPs with the use of aqueous honey solutions in the specified concentration and temperature conditions. It was shown that the higher temperature of 70 °C was necessary to initiate the synthesis of AgNPs in the 20% solution. The lower temperature was sufficient only in the case of the 2% solution. The inhibition of AgNP synthesis in the 20% solution at 35 °C was probably related to the process of protein aggregation, as corroborated by the results obtained with most of the analytic techniques used, particularly RLS.

The AgNPs-H were shown to have higher antifungal activity than AgNPs-C. This may be related to the different zeta potentials of the analyzed colloids. The higher activity of AgNPs-H may also be associated with the presence of flavonoids contained in honey, which have proven antimicrobial activity. The antifungal activity of AgNPs-H was dependent on the concentration of honey. In the microdilution studies, the higher concentrations of honey used for the synthesis of nanoparticles served as a substrate for the fungal strains. The analysis of the inhibition zones showed similar effectiveness of nanoparticles synthesized at the high and low concentrations of honey. This may imply different biomedical applications of AgNPs produced at different concentrations of honey. Further studies focused on the mechanism of action of the ‘green-synthesized’ AgNPs on the fungal cell membrane in honey solutions are advisable.

## 5. Materials and Methods

### 5.1. Chemicals and Reagents

Silver nitrate (AgNO_3_), trisodium citrate (C_6_H_5_O_7_Na_3_), sodium hydroxide (NaOH), and hydrochloric acid (HCl) (Sigma Aldrich, St. Louis, MO, USA) of analytical-grade purity were used as starting materials without further purification. Double-distilled water was used in all experiments.

### 5.2. Citrate Reduction Method

The synthesis of nanoparticles was carried out using a modified Lee and Meisel method [16]. Typically, 50 mL of a 1 mM AgNO_3_ aqueous solution was heated up to boiling temperature, and the solution was stirred vigorously during the synthesis process. While stirring, a solution of 1% sodium citrate (5 mL) was added. The solution was kept at the boiling point for 10 min until the colloids changed color. The solution was heated continuously until the color changes were apparent (pale green). At that point, heat was no longer applied, and the solution was stirred continuously until it cooled to room temperature. The size of the synthesized AgNPs was controlled by the pH of the solution during synthesis.

### 5.3. Honey-Mediated Synthesis

Organic fir honeydew honey was obtained from sources located in the Bieszczady Mountains (Southern Poland) and purchased directly from a beekeeper. Twenty grams of honey were dissolved in 80 g of deionized water, producing a 20% (H-20) solution. Subsequently, two solutions, i.e., 10% (H-10) and 2% (H-2), were obtained by diluting the stock solution. Then, 250 µL of the AgNO_3_ (100 mM) solution was added to 25 mL of each solution described above. To initiate the reduction of Ag^+^ ions, the pH was adjusted to 9.5 using NaOH.

### 5.4. Electronic Absorption Spectroscopy

The electronic absorption spectra were recorded using a double-beam Cary 300 Bio UV-Vis spectrophotometer (Varian, Palo Alto, CA, USA) equipped with a thermostated cuvette holder with a 6 × 6 multicell Peltier block. The temperature was controlled using a thermocouple probe (Cary Series II from Varian) placed directly in the sample. The FWHM (full width at half maximum) was calculated from the fits of the Voigt function to every peak with the use of OriginPro 2020 software.

### 5.5. Fluorescence Spectroscopy

Resonance light scattering (RLS) measurements were performed using a Cary Eclipse spectrofluorometer (Varian). RLS with synchronous scanning at λex = λem (Δλ = 0) from 250 to 850 nm was recorded.

### 5.6. Dynamic Light Scattering Method

Measurements of the intensity-weighted mean hydrodynamic diameter (Z_ave_) [61], electrolytic conductivity, and electrophoretic mobility (laser doppler electrophoresis) [62] were performed by means of Zetasizer Nano ZS (Malvern Ltd., Malvern, UK) in six repetitions at 20 °C. Henry’s equation was applied for calculation of the zeta potential [63]. Additionally, the differential particle size (hydrodynamic diameter) distributions and zeta potential distributions characterizing the synthesized particles were obtained by subtraction of the curve determined for pure honey (or citrate solution) from that describing systems containing AgNPs [24]. This was done to determine the particle diameter or zeta potential at which an increase in the percentage of scattered light intensity occurred after the AgNPs synthesis.

### 5.7. Microorganism and Culture Conditions

The reference strains of *Candida albicans* NCPF 3153 and *Candida parapsilosis* ATCC 22019 were used. The strains were stored at −70 °C in a cryoprotective medium of VIABANK. Before the experiment, the strains were inoculated into a liquid YPD medium (peptone, 20 g/L, yeast extract, 10 g/L, glucose 20 g/L) and cultured for 24 h at 35 °C with shaking. The fungal inoculum was adjusted to yield a concentration of 1 × 10^6^ cell/mL.

### 5.8. Chemical Composition of the Honey

Moisture and extract contents were determined using an Abbe Carl Zeiss refractometer (Germany) on the basis of the refractive index of the honey in its liquid state according to Bogdanov [64]. The pH and electrical conductivity were determined as described earlier [64] using a pIONneer 65 Meter (Radiometer Analytical, Villeurbanne, CEDEX-France) with a combined pH electrode (E16M340) and a four-pole conductivity cell (CDC 30T) with a built-in temperature sensor. Free acidity was determined by potentiometric titration with 0.1 N sodium hydroxide solution (NaOH) and expressed as milliequivalents of acid per kg of honey [64]. The concentration of 5-HMF (5-(hydroxymethyl-)furan-2-carbaldehyde) was determined according to White [65]. The measurement was made with a Carry 300 Bio spectrophotometer (Varian Australia PTY, Ltd., Belrose, NSW, Australia) at wavelengths of 284 and 336 nm, and the results were expressed as mg/kg. Total phenolic content was determined in a reaction with Folin–Ciocâlteu (F–C) reagent according to the procedure described by Singleton and Rossi [66], and the results were expressed as gallic acid equivalent in mg GAE/kg of honey. Total flavonoid content was determined in a reaction with aluminum chloride according to Ardestani and Yazdanparast [67], and the results were expressed as quercetin equivalent (QE) in mg QE/100 g of sample. The chemical composition and physicochemical properties of fir honeydew are presented in Table 3.

### 5.9. Broth Microdilution Method

The broth microdilution method with identification of the minimal inhibitory concentration (MIC) was employed according to the standard methodological guidelines recommended by the Clinical and Laboratory Standards Institute (CLSI), document M27-A3 and M38-A2, for testing the susceptibility of yeasts. The method was modified by addition of 2% glucose to RPMI 1640 medium in order to optimize the fungal growth conditions. To determine the MIC values, an inoculum was prepared with the final density of 0.5–2.5 × 10^3^ cells/mL for yeast, diluted in RPMI 1640 medium, without phenol red and sodium bicarbonate, buffered to pH 7.0 with 0.165 mol/L 3-(*N*-morpholino)-propanesulfonic acid (MOPS) and supplemented with 2% of glucose. The stock solutions of AgNPs were used to obtain the final dilutions in the broth medium on 96-well microtiter plates. The control of fungal growth was performed in medium supplemented with the solvent. After inoculation, the microtiter plates were incubated for 48 h at a temperature of 35 °C. The optical density was determined spectrophotometrically using an EPOCH 2 microplate reader (BioTek, Winooski, VT, USA) at a wavelength of 600 nm. The MIC value was determined by calculating the percentage of the control growth in each replication. The MIC was the lowest concentration of AgNPs capable of 50% inhibition of fungal growth in comparison with the growth of AgNPs-untreated cells.

### 5.10. Disc Diffusion Assay

The antifungal activity was achieved using the Kirby–Bauer disc diffusion method. The disc diffusion tests were carried out to determine the size-selective bacterial sensitivity toward AgNPs, marked by their ZOI. A suspension of all *Candida* strains (0.1 mL of 10^6^ cells/mL) was spread on plates with YPD agar medium (peptone, 20 g/L, yeast extract, 10 g/L, glucose 20 g/L, agar, 20 g/L). Sterile 6 mm paper discs impregnated with the solution of AgNPs (1 μg/disc, 0.5 μg/disc, and 0.25 μg/disc) were placed on the inoculated surface. The test was also performed with AmB (1 μg/disc, 0.5 μg/disc, and 0.25 μg/disc) and fluconazole (1 μg/disc, 0.5 μg/disc, and 0.25 μg/disc). The plates were incubated at 35 °C for 24 h. The results were read after 24 h of incubation. The diameters of the inhibition zones were measured in millimeters.

### 5.11. Statistical Analysis

The tests were carried out in triplicate, and experimental data were reported as the mean ± standard deviation (SD). The differences in antifungal activities of all antifungal agents were compared by ANOVA in OriginPro 2020 software. A *p*-value < 0.05 was considered statistically significant.

## Figures and Tables

**Table 1:** The properties of both the samples after AgNPs synthesis and the initial solutions used.

Sample	Z_ave_ (nm)	PdI	EC (mS/cm)	ZP (mV)
AgNPs-C	18 ± 1	0.65 ± 0.04	0.082 ± 0.002	−43 ± 1
C	-	-	0.276 ± 0.003	-
AgNPs-H2	63 ± 6	0.76 ± 0.12	0.069 ± 0.001	−27 ± 3
H2	382 ± 26	0.46 ± 0.05	0.032 ± 0.003	−28 ± 3
AgNPs-H10	86 ± 27	0.76 ± 0.18	0.120 ± 0.001	−31 ± 1
H10	400 ± 19	0.40 ± 0.06	0.152 ± 0.001	−25 ± 1
AgNPs-H20	106 ± 2	0.96 ± 0.04	0.200 ± 0.001	−28 ± 2
H20	405 ± 12	0.36 ± 0.03	0.252 ± 0.002	−23 ± 1

Z_ave_—the intensity weighted mean hydrodynamic diameter; PdI—polydispersity index; EC—electrolytic conductivity; ZP—zeta potential; the results are shown as the mean value from 6 independent measurements ± standard deviation.

**Table 2:** Minimal inhibitory concentrations (MICs) of silver nanoparticles (AgNPs) against *Candida* strains.

Fungal Strains	MIC (μg/mL)			
	AgNPs-C	AgNPs-H2	AgNPs-H10	AgNPs-H20
*C. albicans*	2	0.5	-	-
*C. parapsilosis*	1	0.5	-	-

**Table 3:** Characteristics of physicochemical properties and antioxidant activity of honey (means ± standard deviations).

Parameter	Fir Honeydew
Moisture (%)	16.42 ± 0.03
Extract (%)	82.08 ± 0.03
pH	4.28 ± 0.03
Free acidity (mEq·kg^−1^)	41.2 ± 0.28
Electrical conductivity (mS·cm^−1^)	1.124 ± 0.005
5-HMF (mg·kg^−1^)	0.61 ± 0.8
Total polyphenols (mg GAE/100 g)	63.25 ± 0.09
Total flavonoids (mg QE/100 g)	9.4 ± 0.07

## Data Availability

Data is contained within the article.
